# Impact of gender on COPD expression in a real-life cohort

**DOI:** 10.1186/1465-9921-15-20

**Published:** 2014-02-17

**Authors:** Nicolas Roche, Gaetan Deslée, Denis Caillaud, Graziella Brinchault, Isabelle Court-Fortune, Pascale Nesme-Meyer, Pascale Surpas, Roger Escamilla, Thierry Perez, Pascal Chanez, Christophe Pinet, Gilles Jebrak, Jean-Louis Paillasseur, Pierre-Régis Burgel

**Affiliations:** 1 Service de Pneumologie et Soins Intensifs Respiratoires, Groupe Hospitalier Cochin Broca Hôtel-Dieu, AP-HP and Université Paris Descartes, Sorbonne Paris Cité, Paris, France; 2 Service de Pneumologie, Hôpital Maison Blanche, CHU de Reims, Reims, France; 3 Service de Pneumologie, Hôpital Gabriel Montpied, CHU Clermont-Ferrand, Clermont-Ferrand, France; 4 Service de Pneumologie, Hôpital Pontchaillou, Rennes, France; 5 Service de Pneumologie, CHU Saint Etienne, Saint Etienne, France; 6 Service de Pneumologie, Hôpital de la Croix-Rousse, Lyon, France; 7 Centre Médical de Bayère, Charnay, France; 8 Clinique des voies respiratoires Hopital Larrey, Toulouse, France; 9 Service de Pneumologie, Hôpital Calmette, Lille, France; 10 Département des Maladies Respiratoires, AP-HM, Université de la Méditerranée, Marseille, France; 11 Clinique des Fleurs, Ollioules, France; 12 Service de Pneumologie, Hôpital Bichat, AP-HP, Paris, France; 13 EFFI-STAT, Paris, France

**Keywords:** COPD, Gender, Clinical expression, Lung function, Age

## Abstract

Reports regarding gender-related differences in COPD expression have provided conflicting results. In the French Initiatives BPCO real-world cohort, which contained 688 patients (146 women) when data were extracted, women were matched with men (1:3 ratio: n = 107:275) on age (5-year intervals) and FEV_1_ (5% predicted intervals) and comparisons were performed using univariate logistic regressions. For a given age and level of airflow obstruction, women with COPD had higher BOD scores due to more pronounced dyspnea and lower BMI, suggesting worse prognosis, and were more likely to exhibit anxiety, suggesting the need for specific assessment and care.

## Introduction

Several analyses of data from observational or interventional studies have been performed to assess gender-related differences in the risk and/or expression of COPD. However, results have been somehow contradictory, some [1-3] but not all [4,5] studies finding more severe expression of COPD in women. Discrepancies might in part relate to differences between studies in terms of genetic background and exposure to risk factors other than cigarette smoking, including occupational or domestic exposures [6,7]. Another explanation could be confounding by severity of airflow limitation [4,8]. To explore whether the clinical expression of the disease is affected by gender in a real-life COPD population, we performed a matched analysis of men and women of the INITIATIVES BPCO cohort. The main goal was to determine whether, for a given age and level of airflow obstruction, clinical features were different in women.

## Methods

COPD subjects included in the present analysis were recruited in the INITIATIVES BPCO cohort between January 2005 and June 2010. INITIATIVES BPCO is an ongoing real-world rolling cohort of spirometry-diagnosed COPD patients (post-bronchodilator FEV_1_/FVC ratio < 70%) identified in 17 pulmonary units of university hospitals located throughout France, which has already been described in details [9,10]. Data are recorded in a standardized case report form but, due to the real-world nature of patient’s care, only demographic characteristics and spirometry are mandatory to include a patient. The study was approved by the Ethics Committee of Versailles (France), and all subjects provided informed written consent.

Women and men were matched (1:3) on age (5-year intervals) and FEV_1_ (5% predicted intervals). Relations between gender and other variables were first analyzed using univariate logistic regressions. To obtain estimates of patients’ prognosis, BOD scores were calculated using Body-Mass Index, FEV_1_ level and MRC dyspnea grade, as previously described [10].

## Results

When the database was extracted, the INITIATIVES BPCO cohort contained data on 688 COPD subjects including 146 women (21%). Altogether, 107 women (73% of the whole female population) could be matched with 275 men (51% of the whole male population). Table 1 presents the characteristics of men and women who were studied.

**Table 1:** **Characteristics of the studied population and univariate comparisons (univariate logistic regression analyses) between age- and FEV**_**1**_**-matched (3:1 ratio) men (n = 275) and women (n = 107)**

**Variable**	**Men**	**Women**	**P value**
Age*	63 [57–71]	63 [56–70]	0.68
Pack-years	41 [25–55]	40 [30–55]	0.65
BMI	25 (22–29)	24 (20–28)	0.02
FEV^1^ (%)*	46 [36–62]	47 [37–65]	0.71
Exacerbations/patient/year	1 [0–2]	1 [0–2]	0.56
Dyspnea (MRC)	1 [1,2]	2 [1-3]	0.0003
SGRQ total	43 [30–59]	46 [32–60]	0.35
BOD index	2 [1-4]	3 [1-4]	<0.0001
Denutrition BMI ≤18.5 kg/m^2^	8%	18%	0.004
Obesity BMI > 30 kg/m^2^	21%	15%	0.18
Comorbid asthma	13%	10%	0.42
Seasonal rhinitis	9%	16%	0.04
Occupational exposure	41%	15%	<0.0001
Chronic bronchitis	68%	70%	0.84
Hypertension	35%	33%	0.71
Left heart failure	10%	15%	0.12
Ischemic heart disease	15%	7%	0.10
Peripheral artery disease	13%	10%	0.58
Diabetes mellitus	14%	9%	0.25
Sleep apnea syndrome	8%	2%	0.04
Anxiety (HAD anxiety ≥10)	29%	41%	0.02
Depression (HAD Depression ≥10)	15%	23%	0.56

*Indicate matching variables. Data are median [Q1–Q3] or %.

Univariate logistic regression analyses found that female gender was associated with a lower frequency of exposure to occupational risk factors, more frequent impairment of nutritional status, higher dyspnea grades and BOD scores and more frequent anxiety (HAD-anxiety > 10) (Table 1). Among comorbidities, sleep apnea syndrome was more frequent in men. Female gender tended to be associated with a more frequent history of left heart failure.

## Discussion

In the present cohort, some components of the clinical expression of COPD, namely BMI and dyspnea, differed between women and men for a given age and FEV_1_ level, leading to higher BOD score in women. Anxiety was also more frequent in women, while HRQOL and exacerbations frequency were not significantly different between men and women. One strength of this study is that matching could be performed using rather small age and FEV_1_ intervals, allowing ensuring that the two populations were similar regarding these variables, as confirmed by univariate comparisons. One limitation is that patients were recruited in pulmonary units of university hospitals and therefore do not represent the COPD population at large. However, it included patients with broad levels of airflow obstruction and multiple phenotypes [9,10], which is infrequent in most randomized trials. One of these, the TORCH trial, which recruited patients with moderate-to-very severe levels of airflow obstruction [4], found that dyspnea and health status impairment were both more pronounced in women despite higher FEV_1_. In the study by de Torres et al., dyspnea was also more intense (while cough was more frequent) in women matched with men on FEV_1_% predicted [11]. Interestingly, these authors found that factors independently associated with dyspnea and HRQL differ between men and women, and suggested that non-respiratory factors may contribute more to dyspnea and HRQL in women [11]. Accordingly, several studies found a higher rate of psychological distress in women [12-14].

Multiple factors could explain gender-related differences in dyspnea ratings, as reviewed a few years ago by Camp and coworkers [15]. Firstly, from a physiological perspective, normal anatomical differences lead to reduced maximal ventilatory capacity, greater expiratory flow limitation, mild decrease in end-expiratory lung volume during exercise and increased work of breathing in women. Secondly, among COPD patients with a given level of airways obstruction, women exhibit more marked airway hyperresponsiveness. Whether this observation relates only to intrinsic differences in airway caliber or also to variations in biological mechanisms is unknown. The affective dimension of dyspnea might also differ between men and women. Finally, hormonal influences could be involved.

## Conclusion

The present study shows that, for a given age and level of airflow obstruction, women with COPD experience different intensity of dyspnea than men. In addition, women have lower BMI, which also contributes to their higher BOD scores. Finally, anxiety appears more frequent in women. Mechanisms underlying these differences remain to be fully understood, but this suggests that COPD assessment and treatment might benefit from a more gender-specific approach.

## Abbreviations

BMI: Body mass index; BOD: Body mass index, (airway) obstruction, dyspnea; COPD: Chronic obstructive pulmonary disease; FEV1: Forced expiratory volume in 1 second; FVC: Forced vital capacity; HAD: Hospital anxiety and depression scale; HAD-A: Hospital anxiety and depression scale-anxiety; HAD-D: Hospital anxiety and depression scale-depression; HRQoL: Health-related quality of life; IQR: Interquartile range; mMRC: Modified Medical Research Council dyspnea scale; SGRQ: St. George’s Respiratory Questionnaire.

## Competing interests

The authors declare that they have no competing interests.

## Authors’ contributions

NR, PRB and JLP designed the analysis plan. Analyses were performed by JLP. All authors contributed to the cohort design, recruitment, data collection, discussion of analysis plan and results, discussion and final approval of the submitted manuscript.
